# Reply to the letter to the editor

**DOI:** 10.1186/s40662-021-00271-1

**Published:** 2021-12-09

**Authors:** Jorge L. Alio, Francesco Versaci, Francesco D’Oria

**Affiliations:** 1 Vissum Miranza, Alicante, Spain; 2 Division of Ophthalmology, Universidad Miguel Hernández, Alicante, Spain; 3 R&D Department, Costruzione Strumenti Oftalmici (CSO), Florence, Italy; 4 Section of Ophthalmology, Department of Basic Medical Science, Neuroscience and Sense Organs, University of Bari, Bari, Italy

We appreciate the interest of Miguel Faria Ribeiro in our paper [1], as well as the comments made to it.

The interest of the topic is sound, as nowadays an enormous amount of new so-called “premium lenses” are appearing in the market, with different optical profiles which obviously lead to different light distributions and different quality of retinal image. This is why our interest in this topic is longstanding and we have published extensive summaries about the outcomes and complications of multifocal lenses over the last years [2, 3]. In these papers, we have clearly defined what we expect clinically from these new technologies, especially from multifocal lenses. However, the issue becomes more important when we try to understand why these outcomes, if not good, are happening. This is the reason why we are exploring a way to analyse the clinical quality of the retinal image in the human eye following the implantation of such innovative optics that multifocal lenses have nowadays. So far, there has been no way to investigate the real quality of retinal image. Optical bench studies do not offer information about the clinical performance of innovative optics, as we know that the human eye is by definition an off-centred optical system and what is obtained on the optical bench does not correspond at all to what is really happening inside the eye at the level of the retina. This is why developing a method in which we can have clinical objective information to compare different types of optics is sound.

In the paper published [1], we have used for the first time pyramidal aberrometry for this purpose. In doing that, we have analysed the optical behaviour of a monofocal lens and apart, a group of multifocal diffractive and refractive lenses, and an accommodative lens which provide different types of performances. Even though we acknowledge the criticism that is raised by the author of the letter about methodology using multifocal lenses, he is already acknowledging that pyramidal aberrometry offers today a much higher level of analysis than previously with the Hartmann-Shack sensors. What Miguel Faria Ribeiro asserted is correct if we had claimed to extend our study to close replicas, something that is not the case. It is well known that an aberrometer is unable to correctly interpret secondary replicas generated by a diffractive lens. However, this is not the case with our work: Indeed, in all the paper it is clearly stated that we are focused on the retinal image in the far focus, and not on the others in which we have less reliability of measurements which generally in this respect is limited.

Bearing this in mind, the outcomes of the monofocal lens obviously cannot be compared to the multifocal ones because we are measuring different levels of light distribution. However, the multifocal diffractive lenses are affected by the same source of bias and, in this way, the IOLs of this group can be compared among themselves. It is the same with refractive lenses, and less affected by the light dispersion that affects the diffractive models and does not affect the accommodative lens analysed in this group and which has been the subject of previous publications [4, 5].

So, we are happy to confirm to Dr. Faria Ribeiro our statements as published in our paper. We thank him for clarifying which light is distributed in the foci along the visual axis in the optical bench, but this is not the topic of our work, as we are dealing for the first time with clinical retinal optical quality in the living implanted human eye. We all already know that the outcomes of the optical bench cannot be extrapolated to the clinical condition of the IOL once implanted in the eye and so we confirm the validity of our experience, and it is the first time that we can analyse, even though probably still partially, the optical quality in terms of PSF, Strehl ratio of different types of lenses implanted in human eyes which have similar levels of corneal aberrations and eliminating the second order according to the methods and technology used here. Clinical quality of retinal image will be an important topic in the future. Further refinement of the methods that are used for this purpose will clarify more in the future which lenses behave properly for visual purposes and will allow the physician to understand and choose the best ones for their surgical practice.

## Data Availability

Not applicable.
